# The influence of psychological needs and motivation on game cheating: insights from self-determination theory

**DOI:** 10.3389/fpsyg.2023.1278738

**Published:** 2023-12-22

**Authors:** Sung Je Lee, Eui Jun Jeong, Dan J. Kim, Jeonyoung Kong

**Affiliations:** ^1^Department of Digital Culture and Contents, Konkuk University, Seoul, Republic of Korea; ^2^Department of Information Technology and Decision Sciences, University of North Texas, Denton, TX, United States; ^3^School of Game, Dongyang University, Pocheon, Republic of Korea

**Keywords:** psychological needs, game cheating, intrinsic motivation, self-determination, autonomy, competence, relatedness

## Abstract

**Introduction:**

Game cheating (i.e., behavior of violating rules in games by using unregulated Software or assistive devices to gain advantage) poses a fatal problem as it destroys fair competition systems and negatively impacts the game ecosystem. Game cheating is reported to be common in competitive online games because they stimulate strongly a user’s motivation and psychological needs. However, there is still in lack of academic research which focused on the issue from the psycho-social perspective.

**Methods:**

This study investigated the relationships between basic psychological needs (i.e., autonomy, competence, and relatedness) and motivation (i.e., intrinsic and extrinsic) based on self-determination theory, and examined their effects on the degree of game cheating with survey data of 322 gamers in a competitive online gaming community.

**Results:**

The results showed the opposite associations between the two forms of motivation (intrinsic and extrinsic) and game cheating. On one hand, extrinsic motivation decreased by autonomy enhanced the degree of game cheating. On the other hand, intrinsic motivation increased by both autonomy and relatedness finally abated game cheating. Competence did not influence any form of motivation. The results indicated that people motivated by interest or enjoyment (i.e., intrinsic motivation) of the game tend to view game cheating negatively while those motivated by game victory and rewards are likely to have positive attitudes toward game cheating. Increasing the degree of user autonomy and social relations in the game could decrease game cheating through the enhancement of intrinsic motivation.

**Discussion:**

Digital game cheating is a crucial problem threatening the spread of game culture and the growth of the eSports industry. The findings of this study reveal the influence of psychological needs and intrinsic motivation related to ‘game cheating,’ providing valuable guidelines in educational and policy aspects.

## 1 Introduction

In recent decades, the digital game industry has rapidly grown. Consequently, however, threats from dishonest gaming behaviors such as game cheating have also increased, especially in online competitive games, the threat of game cheating is much more dangerous. Hence, it is urgent to address this issue. Game cheating can manifest in various ways and forms, and it can even occur in solo play or 1:1 match games. For instance, some players may manipulate videos to make it seem as if they have achieved exceptional performance in speedruns, which are contests aimed at completing games in the shortest possible time. Furthermore, in 2010, in South Korea, a renowned player, Jae-Yoon Ma, became involved in match-fixing during a < Starcraft1> 1v1 tournament, resulting in the forfeiture of several titles and legal penalties (Schöber and Stadtmann, 2022). However, cheating in gaming tends to be particularly pronounced in team-based online competitive games. For instance, many popular online games like Overwatch, League of Legends, and Dota2 adopt genres that focus on interpersonal competition, such as First-Person Shooter (FPS) and Multiplayer Online Battle Arena (MOBA). These online competitive games have systems that provide various rewards, such as badges or emblems representing status, and rare special items to winners to encourage tournament participation. However, the winner-takes-all reward system might feel *unfair* to some users who lack skills, and some may engage in dishonest gaming behaviors to obtain their desired rewards. Additionally, in team-based competitive games, there is a higher likelihood that newcomers may receive negative evaluations from skilled teammates or face taunting from winners when their team loses (Lee et al., 2019; Tan and Chen, 2022). With a heightened likelihood of encountering such verbal aggression, individuals may resort to cheating as a means to protect their reputation and achieve victory more easily.

Cheating in online competitive games poses a fatal problem as it threatens fairness, a fundamental premise of the tournament (Lee et al., 2021). Rampant cheating reduces trust in the competition system, diminishes the inherent fun of the game, leads to player dropout, and inflicts serious damage to the game company’s brand image (Yan and Randell, 2009; Wu and Chen, 2018). For example, globally popular games like League of Legends, Overwatch, and Valorant have faced criticisms and skepticism from the gaming community in dealing with cheating issues, such as illegal software use and glitching (bug abuse). Some games have even incurred severe damage since many players refused to participate and experienced intensified desocialization (Lee, 2016; Harrison, 2022; Noah, 2022). In addition, cheating scandals in eSports leagues in France, North America, and Korea negatively impact public perception of the game leagues and professional players and in the long term, have also damaged the development of the eSports industry (David, 2014; Nordmark, 2018). In this context, rampant game cheating should not be underestimated as a temporary and trivial deviation but should be interpreted as a serious threat that destroys fair competition systems and negatively impacts the game ecosystem. Accordingly, game companies are constantly investing resources and workforce to develop security programs that can detect and prevent cheating in advance, and take post-action measures such as ‘bans’ (Noah, 2022). In academia, studies on technical countermeasures and methods to detect and prevent online game cheating are conducted (Han et al., 2022; Qian et al., 2022), as well as research aiming to discover psychosocial factors influencing game cheating and potential countermeasures (Vorderer et al., 2004; Lee et al., 2021; Schöber and Stadtmann, 2022). However, research is still primarily focused on providing solutions from a technical perspective, and there is a lack of studies understanding game cheating users and establishing countermeasures from a socio-psychological perspective, regardless of the seriousness of game cheating and the importance of analyzing the psychological characteristics and motivations that lead users to dishonest gaming behaviors and establishing strategies to prevent them.

Game cheating tends to be more common in games with competitive structures than in non-competitive games. This is because players participating in games with competitive contexts are more likely to be motivated by extrinsic factors such as competitive victories and rewards than other intrinsic factors (Vorderer et al., 2004). Social pressures and contexts emphasizing competitive victories can weaken a player’s psychological resistance to cheating (Lee et al., 2021). For instance, rare rewards, identifiable items that can flaunt victories, and the distorted community atmosphere and feedback that solely prioritize victory can act as catalysts, fostering the player’s misguided desires for victory (Rigdon and D'Esterre, 2015; Cruz et al., 2017; Wu and Chen, 2018). Therefore, understanding the psychological desires and motivations that lead players to cheat in a competitive environment is vital. Another important fact to consider is that team-based online competitive games can cater to various individual desires, such as voluntary participation, demonstrating competence, and establishing smooth relationships with teammates. Moreover, individual gaming behaviors can vary significantly depending on these personal motivations. In other words, while some players may seek pure enjoyment from the game, others may engage in it with the expectation of obtaining rewards and honor. In this regard, self-determination theory provides a useful framework for explaining the influence of fulfilling the needs and various types of motivation on an individual’s task performance behavior (Deci and Ryan, 2000, 2008). In other words, examining players’ diverse fundamental psychological needs and motivational levels through the framework of self-determination theory can be beneficial in comprehending misconduct in online gaming.

Self-Determination Theory is a macro theory of human motivation that addresses issues, such as personal character development, self-regulation, and the goals and behaviors for life or specific activities (Deci and Ryan, 2008). The theory assumes that personal traits and social environments influence an individual’s basic psychological needs, the deprivation or fulfillment of these needs stimulates various types of motivation, and this motivation influences an individual’s task setting and the behavior and perception for performing them (Deci and Ryan, 2009). Therefore, self-determination theory has been used to explain how types of motivation in a competitive environment that includes achievement goals, rewards, and evaluations can trigger behaviors, such as pro-social behavior, anti-social behavior, and cheating (Ntoumanis and Standage, 2009; Kanat-Maymon et al., 2015; Mallia et al., 2019). However, studies that examine game cheating through self-determination theory are relatively rare. Accordingly, this study looked at the relationship between basic psychological needs, extrinsic motivation, and intrinsic motivation as the main concepts of self-determination theory and cheating in digital games using survey data collected from 322 people in an online gaming community.

## 2 Theoretical background

### 2.1 Online game cheating

Cheating shares a common ground with gamesmanship and disruptive behavior as it is a goal-directed behavior for unfair gains. However, it is distinguished by the premise of blatant rule violation (Mallia et al., 2019). Game cheating refers to the behavior of violating rules by using unregulated software and assistive devices or exploiting malicious bugs to gain unfair advantages in certain situations (Schöber and Stadtmann, 2022). Online game cheating has been rampant since the invention of video games, and its severity and threat are growing daily as the anonymity of games is strengthened by the development of internet technology (Wu and Chen, 2013). Game cheating takes various forms, including but not limited to unauthorized changes to the client or hacking, utilization of unauthorized software like Map-hack, Wall-hack, Aim-Hack, Auto-targeting, and Distributed Denial of Service (DDoS), exploiting another player’s misplaced trust, glitching, and elo-boosting (Yan and Randell, 2009; Ghoshal, 2019; Lee et al., 2021). Moreover, recently, drugs like Adderall, which is used to treat attention deficit hyperactivity disorder (ADHD) to improve focus, are also viewed as a type of game-cheating behavior (Schöber and Stadtmann, 2022).

Game cheating undermines the ethical values and fun that competition holds in games, and it also diminishes players’ trust and expectations for fair competition. Competitive elements are crucial components of games, and indeed, many digital games have goal-oriented and competitive contexts (Song et al., 2013). In this regard, Huizinga (2014) referred to the fun that comes from serious competition ensured by fairness as ‘agon’ and presented it as one of the fundamental attributes of games and play. In this context, victory in competitions can extend to various forms of value not confined to the game’s outcome, such as proving personal excellence. For example, competition in games can hold greater meaning and value in testing personal limitations (like mastering avatar control or deploying high-level strategies) and demonstrating and learning moral capabilities (Serrano-Durá et al., 2021). Also, the competition itself can be an interesting element that evokes intrinsic motivation (Song et al., 2013). However, game cheating pushes aside the ethical significance and intrinsic value of game competition in acquiring external values, such as rewards or honor. This is because game cheating allows only specific players to monopolize potential benefits from competitive victory unfairly (Chen and Ong, 2018).

In particular, game cheating can be more detrimental in team-based online competitive games. Cheating in solo-play based eSports is primarily associated with issues of record-keeping, tournament trustworthiness, and rewards. However, cheating in team-based online competitive games can impact not only fairness and rewards but also disrupt the enjoyment and positive emotions of those participating in the game, as well as undermine gaming community norms. For instance, if there is even one cheater, most regular players matched in that game must give up the game or endure psychological discomfort while continuing an unfair game. In this process, the motivation and intention of other players who want to feel the inherent fun of game competition or prove their excellence through a fair victory are inevitably excluded, and the fair competition opportunities that should have been given to the players are ‘robbed’ by external forces. Considering that ‘agon’ occurs based on fair competition (Huizinga, 2014), the prevalence of game cheating that threatens the fairness of the competitive structure can be said to be fatal to the fun of the game and its normal operation. If such a situation continues, game players may distrust the institutions or publishers responsible for ensuring fair competition, or they may demand aggressive countermeasures, which could lead to de-socialization, where they leave the game community where cheating is rampant (Yan and Randell, 2009; De Paoli and Kerr, 2012; Wu and Chen, 2013).

Game cheating can also harm game publishers and developers, not just players and gaming communities. Game publishers and developers need to invest a significant amount of money and workforce in developing anti-cheating and detection programs to stabilize the game system and prevent user attrition. A cheating scandal in one eSports league shows that the problems caused by game cheating are not only a problem for the players but also for various entertainment sports areas based on the game (David, 2014; Nordmark, 2018). However, despite these risks, young gamers are less likely to identify unfair practices in online virtual environments as ‘ethical problems,’ and there is even a risk of accepting them as behavioral norms in situations where unfair practices are uncontrolled (Wu and Chen, 2018).

Some game cheating methods are difficult for an average player to carry out, as they require specialized knowledge of the software. However, other ways of cheating are relatively easily accessible. For instance, techniques such as glitching, once discovered by chance, can easily spread through game communities or streaming services and continue until the developers patch or hotfix the issue. Some players may look up information related to cheating programs or purchase products through websites and forums (Hamlen and Gage, 2011), while some may even hire top-tier players to boost their rankings (i.e., elo-boosting) (Sacco, 2017). These facts indicate that the threat of game cheating is diverse and widespread and can occur far more frequently than expected. Countermeasures like anti-cheating programs may be temporarily effective against certain types of cheating; however, they have limitations, such as provoking hackers to deactivate the anti-cheat or encouraging other types of cheating. Thus, concurrently investigating the paths and psychosocial factors leading to game cheating and developing macro-level response strategies are necessary. According to prior research, the occurrence of game cheating is largely influenced by individual psychological characteristics and motivations in the competitive environment (Vallerand et al., 1986; Lee et al., 2019, 2021; Chen et al., 2022). Motivation toward winning or rewards has particularly been reported as a critical factor in predicting unfair behavior (Vallerand et al., 1986; Lee et al., 2021). Some studies have also suggested that an excessively competitive environment can weaken intrinsic motivation for activities (Vallerand et al., 1986). Others have shown that such an environment can lead to an obsession with winning or rewards, which can influence destructive gaming behavior or cheating when frustration due to failure arises (Ballard and Welch, 2015; Lee et al., 2019, 2021). Hence, this study integrates self-determination theory to understand human behavior changes based on basic psychological needs and types of motivation (Deci and Ryan, 2008) and eventually counter online game cheating.

### 2.2 Self-determination theory and basic psychological needs

Self-determination theory is a macro-motivation theory applicable in various fields, such as personality development, self-regulation, physical activity, and virtual worlds. It is actively utilized in applied research fields, such as education and sports (Deci and Ryan, 2008; Deci et al., 2017). The theory assumes humans as entities whose intrinsic functioning can be activated or impeded by the social context (Deci et al., 1994). Moreover, it emphasizes that motivation is not a single concept, and the type and quality of motivation stimulated in individuals are more important in predicting meaningful behavior and outcomes (Deci and Ryan, 2008). For instance, some people participate in specific activities for the sake of participation itself or the interest or enjoyment it brings, thus, maintaining their intrinsic motivation. Those experiencing this kind of autonomous motivation feel self-endorsement and volition about their choices and activities and continue to engage in them (Deci and Ryan, 2008). However, others might join certain activities for social usefulness or specific goals, even if they are not interested. Those subject to controlled motivation are more likely to be driven by the pressure to conform to a certain way rather than think for themselves or decide about their own activities. This fact signifies that autonomous motivation and controlled motivation contrast with amotivation, which means a lack of intent and vitality for specific behaviors. It also suggests that these motivations facilitate or maintain human behavior and thought in their ways (Deci and Ryan, 2008; Ryan and Deci, 2017).

The types and levels of synchronization can vary depending on individuals’ psychological needs and environmental factors, and they can influence self-determined behavior toward specific activities (Deci et al., 1994). Self-determination theory assumes that individuals’ motivation for specific activities can be influenced by their basic psychological needs (Deci et al., 2017). Deci and Ryan (2008) have reported that individuals have fundamental psychological needs that must be satisfied to achieve effective functioning and psychological health in relation to specific activities, and these needs are universally observed regardless of cultural influences, such as collectivism or individualism. Furthermore, needs such as autonomy, competence, and relationships have been identified as essential in explaining the formation of intrinsic motivation (Sheldon et al., 2003; Baard et al., 2004; Dysvik et al., 2013). In this context, *basic needs* are theoretically distinguished from the everyday usage of the term *needs* (Krapp, 2005). *Basic needs* refer to the most universal form inherent in psychological behavioral motives, which serve as essential prerequisites for personal satisfaction and achievement, the realization and maintenance of one’s potential, and protection from maladaptive functioning (Deci and Ryan, 2002; Ryan and Deci, 2002; Krapp, 2005).”

Autonomy refers to the sense of control or ownership over one’s actions (Wang et al., 2008; Deci et al., 2017). More specifically, autonomy tends to seek the causes of one’s behavior within oneself and the desire to perceive oneself as free from negative internal or external pressures, enabling the choice of specific activities (Dysvik et al., 2013). Previous research suggests that autonomy is one of the most prominent needs among various motivations, and autonomous internalization of a specific activity significantly affects the formation of intrinsic motivation (Deci and Ryan, 2000; Ryan and Deci, 2006; Vallerand et al., 2008; Wang et al., 2008; Dysvik et al., 2013). For example, individuals who choose and engage in specific activities based on their own needs and preferences are more likely to be motivated by their interest in the activity itself and the resulting enjoyment rather than short-term benefits based on performance outcomes. In other words, higher autonomy promotes greater intrinsic motivation for a specific activity (Gagné and Deci, 2005). On the other hand, when the choice of an activity is driven by external factors such as social pressure or rewards rather than one’s own volition, individuals are more likely to engage in the activity to achieve goals or obtain rewards rather than experiencing intrinsic interest in the activity itself. On the contrary, individuals with lower autonomy are more likely to be influenced by goal attainment or reward achievement rather than being motivated by the inherent enjoyment of and engagement in the activity. Thus, a high level of autonomy can activate intrinsic motivation by directing attention to values such as interest or entertainment derived from the external activity itself.

Competence refers to the mastery or efficiency experienced in skills, abilities, or proficiency required to achieve desired outcomes (Deci et al., 1991). In other words, competence signifies the need for a sense of capability, a positive perception of effectively performing a specific activity, and the tendency to demonstrate and express it (Ryan and Deci, 2002). Positive self-perception of competence promotes intrinsic motivation by encouraging engagement in challenging activities, utilizing skills, and sustaining participation in activities (Dysvik et al., 2013). Concerning this, the cognitive evaluation theory (CET), a sub-theory of self-determination theory, suggests that the development of intrinsic and extrinsic motivations occurs progressively through individuals’ perception and evaluation of their competence (Deci and Ryan, 1985; Guay et al., 2001). According to CET, competence can be enhanced or diminished through feedback processes related to the activity’s process or outcome, and this process can also influence the formation and absence of intrinsic motivation (Harackiewicz and Sansone, 1991; Elliot et al., 2000). For example, exceptional performance can positively influence an individual’s sense of competence, strengthening intrinsic motivation. On the other hand, individuals who experience negative outcomes may devalue the worth of their efforts as they perceive it to be unimportant or attribute it solely to external factors (e.g., incentives) to protect their self-esteem or self-worth (Elliot et al., 2000). In line with this, other studies claimed that individuals with higher levels of perceived competence are more likely to activate intrinsic motivation for a specific activity, whereas individuals whose perceived competence is undermined by negative feedback, for example, may experience a decrease in motivation (Fisher, 1978; Deci and Ryan, 1985; Guay et al., 2001). Furthermore, another study conducted with 360 Spanish university students reported the static influence of autonomy and competence on intrinsic motivation (Buil et al., 2019). However, contrary to the results of most studies indicating a static relationship between competence and intrinsic motivation, some minority studies have reported that, unlike relatedness or autonomy, competence does not significantly influence intrinsic motivation (Dysvik et al., 2013).

Relatedness refers to the connection with others or a sense of belonging and mutual care within a group or community (Ryan and Deci, 2002). High levels of social relatedness provide a “formidable opportunity” for fulfilling psychological needs and evoke perceptions of a stable and supportive interpersonal environment (Vallerand et al., 2008; Van Den Broeck et al., 2008). For instance, a sense of belonging to a community with shared interests and needs positively influences one’s well-being and physical and mental health, while frustration with relatedness needs can lead to imbalances in psychological health and negative effects on the formation of intrinsic motivation (Ryan and Deci, 2000b; Krapp, 2005). In this regard, attachment theory explains that positive relationships and securing attachment with others are necessary for individuals to explore their environment with stability (Lopez and Brennan, 2000; Dysvik et al., 2013). This suggests that the likelihood of intrinsic motivation formation is greater within stable relational contexts (Deci and Ryan, 2000). Multiple studies supported the importance of relatedness in forming intrinsic motivation. For instance, a study conducted with 374 Chinese Massive Open Online Courses (MOOC) users found that the satisfaction of basic psychological needs positively impacted intrinsic motivation, and the quality of relationships predicted students’ psychological engagement and facilitated participation in MOOC usage (Sun et al., 2019). Furthermore, a mixed-methods study conducted with early childhood teachers reported that relationships with co-workers and adequate support are crucial predictors of intrinsic motivation formation (Wagner and French, 2010). In this context, while relatedness is sometimes considered a peripheral need compared to competence and autonomy, it has been emphasized as an equally focal aspect in forming intrinsic motivation (Dysvik et al., 2013). However, research findings regarding the relationship between relatedness and extrinsic motivation formation are somewhat inconsistent, and some studies have reported that satisfaction with relatedness does not influence extrinsic motivation (Matsumoto and Takenaka, 2022).

The fact that basic psychological needs influence individuals’ motivation and well-being also applies in online environments. For example, research on the inclination to share innovative information in online gaming communities found that intrinsic motivation positively influenced knowledge sharing, while extrinsic motivation such as status enhancement or rewards negatively affected the knowledge sharing of innovative users in the community (Hau and Kim, 2011). Additionally, other previous studies argued that autonomy, competence, and relatedness are all statically related to game enjoyment, which is linked to intrinsic motivation (Przybylski et al., 2010). Furthermore, this research suggests that higher levels of satisfaction with basic psychological needs are associated with a greater likelihood of participating in games harmoniously. These findings are supported by other studies indicating that the enjoyment of video game usage is maximized when all three basic psychological needs are fulfilled (Tamborini et al., 2010). These results indicate that the influence of basic psychological needs on individuals’ motivation is consistent in offline and online environments.

According to prior research, one of the critical attributes of play and games is the pursuit of enjoyment through autonomous participation (Huizinga, 2014). Conversely, in-game activities like ‘gold farming’ or ‘botting,’ where autonomy is substantially restricted, are more akin to labor than to play or leisure (Dyer-Witheford and De Peuter, 2009). In this context, it can be stated that gaming engagement is inherently and deeply connected with autonomy. However, the level of autonomy can differentially influence motivations and usage patterns, depending on the game’s specific context. For instance, an empirical study reported that in-game customization has a positive impact on autonomy, and higher levels of autonomy correlate with an increase in both enjoyment and a sense of physical presence (Kim et al., 2015). On the other hand, team-based online competitive games are equipped with elements that are conducive to satisfying the need for competence. For example, in these types of games, the likelihood of winning increases as players become more adept at handling game-specific skills, such as intricate character control. Additionally, everyone participating in the game has the opportunity to appreciate and discuss a player’s exceptional skills and accomplishments. Moreover, the games offer various rewards like scores, special items, and badges to players who achieve victory or demonstrate outstanding performance. These elements not only stimulate players’ needs for competence but also influence their perception of their own abilities. In a similar vein, gaming engagement could also be related to ‘relatedness,’ and this concept could be equally applicable to online competitive games that use random matchmaking systems. For example, some players may engage in such games for social reasons, aiming to strengthen their relationships with friends. Conversely, others may play these games to enjoy the process of interacting and competing-cooperating with strangers. In line with this, a study conducted on 132 Taiwanese adolescents reported that basic psychological needs such as autonomy, competence, and relatedness serve as predictors for ‘game playfulness’ (Chiang and Lin, 2010).

### 2.3 Intrinsic motivation and extrinsic motivation

Motivation is the internal drive that guides, directs, and sustains human behavior (Singh, 2011). As research on motivation has accumulated, various theories and concepts have emerged. However, at least in the early studies, motivation was assumed to exist in two types: intrinsic motivation and extrinsic motivation (Alexandris et al., 2002). Even today, these two types of motivation are considered key factors influencing human behavior in domains such as academics, leisure activities, and sports (Howard et al., 2020).

Intrinsic motivation refers to engaging in behavior driven by the enjoyment, fun, pleasure, and satisfaction derived from an activity (Ryan and Deci, 2017). In other words, intrinsic motivation is driven by voluntary interest, curiosity, enjoyment, and exploration without external rewards and can be considered the purest form of autonomous motivation (Gagné and Deci, 2005). Intrinsically motivated individuals are known to pursue novelty and challenges, expand their capacities, and exhibit a more exploratory tendency (Ryan and Deci, 2000b). It is important to note that intrinsic motivation is formed by one’s interest, curiosity, and expectations of enjoyment in a specific activity. For example, a study reported that individuals who experience pleasure in competitive activities are relatively more likely to develop intrinsic motivation for competitive games, whereas those who do not find interest in the competitive elements struggle to form intrinsic motivation even when playing the same competitive games (Song et al., 2013). This suggests that even in competitive contexts where the possibility of motivation through external factors, such as rewards and prestige, is high, individuals can feel interested in the enjoyment of competition over external factors, forming intrinsic motivation. Satisfying basic psychological needs is essential for maintaining and enhancing intrinsic motivation (Ryan and Deci, 2017). According to CET, social contexts that enhance competence, such as acquiring new skills or receiving positive feedback, strengthen intrinsic motivation, while factors that diminish autonomy, such as significant external rewards or behavioral control, can weaken intrinsic motivation (Deci, 1975; Deci et al., 1999). This finding is supported by empirical research indicating that the satisfaction of basic psychological needs activates intrinsic motivation, enjoyment, and intention to continue using games (Ryan et al., 2006).

Extrinsic motivation, on the other hand, refers to engaging in behavior for reasons external to the activity itself. However, it does not mean that all forms of extrinsic motivation completely lack autonomy (Ryan and Deci, 2000a). The spectrum of extrinsic motivation ranges from external regulation, where the cause of behavior is solely placed on external factors, such as rewards or constraints, to introjected regulation, where behavior is driven by self or others’ approval; identified regulation, where behavior is aimed at achieving self-set goals; and integrated regulation, where the cause of behavior is tied to the confirmation of one’s values (Gagné and Deci, 2005). This indicates that extrinsic motivation can exhibit slightly different patterns depending on the relative degree of autonomy. Other studies reported that even within the category of extrinsic motivation, those closer to external regulation or introjected regulation driven by rewards or social approval are more likely to be associated with negative outcomes, such as dropping out or low psychological well-being (Vallerand and Blssonnette, 1992; Sheldon and Kasser, 1995; Ryan and Deci, 2000a). However, extrinsic motivation is still distinct from intrinsic motivation since it does not place the cause of behavior within the activity itself but considers the activity as an instrumental means. In this regard, extrinsic motivation encompasses all instrumental behaviors (Deci et al., 2017).

Reinforcing extrinsic motivation is closely related to the lack of basic psychological needs. A decrease in perceived autonomy can shift the focus from voluntary participation in the activity itself to secondary gains from the activity, particularly tangible rewards and honors. This is supported by research findings that high levels of autonomy negatively impact external regulation (Sheehan et al., 2018). Relatedly, other research has suggested that fulfilling basic psychological needs can transform extrinsic motivation into a self-determined form (Ryan and Deci, 2000a).

In everyday life, the satisfaction or frustration of basic psychological needs can influence an individual’s interest, preference, and motivation in media activities (Kardefelt-Winther, 2014; Fernandez de Henestrosa et al., 2023). For instance, according to the model of compensatory internet use, individuals who suffer from a deficiency in real-life needs may excessively engage in media usage that is expected to satisfy specific needs or may use media in ways that better fulfill their needs. In this context, players who experience a deficiency in basic psychological needs in daily life are hypothesized to be motivated toward games in ways that better satisfy their needs through compensatory mechanisms. For example, users who lack or are deficient in specific needs may find game genres emphasizing competition more appealing (Fernandez de Henestrosa et al., 2023), and in a similar context, they may also show differences in attitudes and patterns of motivation toward competition.

In this contexts, intrinsic motivation and extrinsic motivation are known to be deeply related to sustained game usage and experience in online competitive games. For example, a prior study revealed that high levels of intrinsic and extrinsic motivation are crucial variables in predicting game mastery (i.e., the levels reached), daily usage time, and gamer loyalty (Dindar, 2018). In the case of online competitive games, a blend of direct competition between individuals and features known to enhance intrinsic motivation, such as indirect competition (e.g., online scoreboards), exists, leading to a complex pattern of motivation (Deci and Ryan, 1985). For instance, some people may experience activated intrinsic motivation and enjoyment when they feel they have sufficiently exerted their capabilities, regardless of winning or losing. On the other hand, those who perceive competition as a means for external rewards may be more sensitive to defeat, yet derive greater satisfaction from victories and achievements (McAuley and Tammen, 1989; Sepehr and Head, 2018).

Previous research on the Self-Determination Theory supports the notion that basic psychological needs and motivation play crucial roles in explaining academic or sports misconduct. However, attempts to verify this in the context of digital gaming have been remarkably scarce. In light of this, the present study aimed to investigate whether players’ basic psychological needs influence intrinsic and extrinsic motivations in gaming, and in turn, if these affected motivations shape attitudes toward cheating in games. Based on these points, the following hypotheses have been proposed regarding the relationship between basic psychological needs and intrinsic and extrinsic motivation in games:

H1: As autonomy increases, intrinsic motivation increases (H1a), but extrinsic motivation will decrease (H1b).

H2: As competence increases, intrinsic motivation increases (H2a), but extrinsic motivation will decrease (H2b).

H3: As relatedness increases, intrinsic motivation increases (H3a), but extrinsic motivation will decrease (H3b).

The hypotheses mentioned above were formulated in a manner that generally supports prior research on basic psychological needs and motivation. However, considering the characteristics of team-based online competitive games, different outcomes may emerge, deviating from general trends. Specifically, considering the emphasis on random matchmaking in online competitive games, ‘relatedness’ may not necessarily suppress extrinsic motivation but rather have no significant impact at all. For instance, a high level of relatedness could influence the formation of emotional bonds with randomly matched teammates and contribute to enjoying the game. However, due to the structural features of the game that involve random matchmaking and “competition,” the influence of social bonds and relatedness on the formation of extrinsic motivations like rewards or superior victories may be somewhat limited. Despite some prior research supporting the conjecture that the influence of relatedness on extrinsic motivation may be limited (Huhtiniemi et al., 2019; Matsumoto and Takenaka, 2022), the hypotheses have been put forth with reference to more generalized research findings and theoretical reviews.

### 2.4 Self-determination theory and cheating

According to self-determination theory, extrinsic motivation can weaken psychological resistance to potential misconduct by emphasizing external factors such as rewards rather than intrinsic interest or satisfaction in the task. In line with this, one study reported that extrinsically motivated children, when faced with problematic situations, pay more attention to external evaluations and rely less on self-directed efforts than intrinsically motivated children (Boggiano, 1998; Guay et al., 2001). This suggests that extrinsically motivated individuals prioritize maintaining self-evaluation or acquiring rewards, and when confronted with threatening situations, they may be more inclined to choose a more certain approach, even if it involves some level of dishonesty, rather than relying on their efforts to resolve the issue.

In this context, self-determination theory is also utilized to explain an individual’s prosocial behaviors, rule-breaking, and social behaviors such as cheating. Research in various fields, such as sports and academics, has identified basic psychological needs and motivation as significant factors in predicting or explaining prosocial behaviors and antisocial behaviors, including cheating (Ntoumanis and Standage, 2009; Kanat-Maymon et al., 2015; Mallia et al., 2019). From the self-determination theory’s perspective, the reasons individuals engage in specific behaviors (i.e., their motivational orientation) can also influence their approach to sports and their behavioral patterns (Vallerand and Losier, 1994). For example, enjoyment or social satisfaction can promote reciprocal relationships with others and encourage compliance with rules. A study on the prosocial behavior of adolescent athletes highlighted the influence of fulfilling psychological needs, including autonomy, in promoting the development of moral attitudes and inhibiting antisocial behaviors (Mallia et al., 2019). Furthermore, another study involving 147 master athletes reported a positive correlation between high levels of autonomous motivation and prosocial behaviors toward teammates and opponents (Sheehy and Hodge, 2015).

On the other hand, individuals who engage in certain behaviors due to external rewards or punishment avoidance are more likely to be drawn to misconduct or gamesmanship than those who are not motivated by such external factors (Ntoumanis and Standage, 2009). This finding is supported by other studies showing that intrinsic motivation is positively related to prosocial behaviors, while extrinsic motivation is associated with antisocial behaviors (Hodge and Lonsdale, 2011; Sheehy and Hodge, 2015). Attempts to explain misconduct through self-determination theory are not limited to sports but are also evident in other domains, such as academics. A literature review study revealed that academic cheating is positively related to controlling motivations toward external factors such as material incentives, gaining social approval, and securing good job prospects, while autonomous motivation is negatively associated with cheating (Pulfrey et al., 2019). Furthermore, research on academic dishonesty, including cheating, found that the frustration of psychological needs enhances academic dishonesty, while need satisfaction reduces it (Kanat-Maymon et al., 2015). The same study also reported a positive correlation between perceived need satisfaction and autonomous motivation was negatively correlated with academic dishonesty.

From this perspective, winning in online competitive games can be considered a factor triggering extrinsic motivation. Particularly in games, winning not only relates to personal achievement but also has a significant impact on external evaluations of the individual and the acquisition of specific rewards. For example, high game scores achieved through cumulative victories in game communities are utilized as important criteria for evaluating an individual’s skill. “High game scores and rankings,” especially among younger generations of gamers, can be regarded as measures of honor and display in themselves. For example, a prior study pointed out that game-proficient adolescents intentionally ‘present’ or ‘boast’ about their exceptional achievements to peers, thereby eliciting envy mixed with admiration relationships (Seo and Lee, 2017). And in anonymous gaming environments where identity information is limited, rewards can serve as significant indicators that confer authority over an individual’s speech or actions (Cruz et al., 2017). In other words, winning itself provides benefits for improving an individual’s reputation or external evaluations within the game community.

On the other hand, winning stimulates extrinsic motivation through the mechanism of rewards. For instance, many online competitive games provide rewards, such as limited-edition items or special titles, to individuals who achieve high scores and ranks, aiming to encourage users’ competitive participation (Lee et al., 2021). These reward systems are designed with a differentiated distribution method where the rewards become rarer and more appealing as the ranking gets higher. Therefore, users who are motivated by rewards are more likely to expect and strive to accumulate points easily and abundantly. Moreover, if the value and magnitude of the rewards (such as rarer and more attractive items or larger prize money) outweigh the costs associated with cheating in the game or the potential losses from being caught, players may perceive dishonest gaming behavior as a superior choice (Schöber and Stadtmann, 2022). Consequently, excessive interest and obsession with winning or achievements can mitigate psychological resistance to dishonest behavior in competitive contexts and environments (Whitley, 1998; Rigdon & D’Esterre). This fact is supported by other studies showing that the higher the expected value and significance of rewards, the greater the value attached to dishonest behavior (Ballard and Welch, 2015; Cruz et al., 2017; Wu and Chen, 2018). For example, according to research on academic dishonesty among Korean university students, goals related to intrinsic motivation, such as self-growth, negatively predict minor and serious cheating, while motivation related to rewards, such as wealth, only shows a positive correlation with minor cheating (Park, 2020). Additionally, other studies have found that in game reward systems, when extrinsic motivation is enhanced, intrinsic motivation and enjoyment are weakened, and the likelihood of engaging in in-game cheating decreases as the perceived benefits of rewards diminish (Cruz et al., 2017; Wu and Chen, 2018). Therefore, in competitive situations, a fixation on victory, encompassing honor, self-display, and rewards, might cultivate a positive attitude toward cheating and further encourage dishonest behaviors (Ballard and Welch, 2015; McInroy and Mishna, 2017; Lee et al., 2021). In relation to this, a prior study has shown that extrinsic motivation and a positive attitude justifying academic dishonesty have a direct effect on cheating behaviors, with the influence of attitude being even more significant (Jordan, 2001).

Based on these findings, the following hypotheses are proposed:

H4: Intrinsic motivation will be negatively related to attitudes about game cheating.

H5: Extrinsic motivation will be positively related to attitudes about game cheating.

## 3 Method

### 3.1 Procedure and participants

League of Legends is an online game globally popular and serviced worldwide, such as in China, Europe, and North America. It is particularly popular in South Korea. In this context, the researchers recruited participants from the player base of League of Legends, a representative competitive-cooperative game in the online gaming industry. The survey participants were recruited from large League of Legends communities in South Korea (e.g., lol.inven.co.kr). The participants were provided with a hyperlink to a webpage containing information about their rights as research participants and privacy protection. Only those who read and agreed to participate in the study were directed to an online survey constructed on the survey platform SurveyMonkey (www.surveymonkey.com). Participants were limited to those with recent experience with League of Legends within the past month. The recruitment process took approximately 3 days, and participants were given mobile culture vouchers (approximately 3 USD) as compensation for their participation. The questionnaire included questions about sex, age, and average daily game usage time, as well as research scales. This study was conducted with the approval of the university’s Institutional Review Board (IRB).

### 3.2 Data analysis

This research paper used SEM (Structural Equation Model) to analyse the model. SEM was also used to consider the hypotheses and relationship among variables. In this study for describing data and other results was used SPSS 22 0 and for examining goodness of fit of model was used Amos-22.

A total of 362 responses were collected, but data from 40 participants were excluded due to factors such as long-string responding and premature termination, indicating careless responding. Therefore, 322 data sets were utilized for the study. The participants included 226 males (70.2%) and 96 females (29.8%), with an average age of 28 years (SD = 5.03). Among them, the largest age group was in their 20s, with 172 participants (53.4%), followed by the 30s with 132 participants (41%), and the 10s with 14 participants (4.3%). The smallest group was those in their 40s or older, with only 4 participants (1.2%). The average daily game usage time, measured in 30-min intervals (1, less than 30 min – 10, more than 300 min), was approximately 99.56 min (SD = 48.89 min). Most male users were in their 20s with 129 participants (57.1%), followed by the 30s with 83 participants (36.7%), the 10s with 10 participants (4.4%), and the 40s with 4 participants (1.8%). The average daily game usage time for male users was 101.61 min (SD = 50.79). Among female users, the majority are in their 30s with 49 participants (51%), followed by the 20s, with 43 participants (44.8%), and the 10s with 4 participants (4.2%). The average daily game usage time for female users was 94.69 min (SD = 43.961).

### 3.3 Measurements

We used four items based on Lee et al. (2021) to measure attitude about game cheating. The items measure players’ perceptions and attitudes toward game cheating. For example, each item was formulated as “I do not think it is bad to play bugs or hacks,” “For fun, I think it is okay to use a hack or glitching play,” and “For a win, I think it is okay to use a hack or glitching play.” Responses ranged from 1 (strongly disagree) to 5 (strongly agree).

Then, we used a subset of the Basic Psychological Needs Scales (BPNS) scale (La Guardia et al., 2000) to measure basic psychological needs. The BPNS scale assesses the satisfaction of needs in general life or specific domains. It consists of sub-factors, such as autonomy, competence, and relatedness (see Table 1). We utilized nine items, with three items for each sub-factor. The autonomy items included statements such as “There is not much opportunity for me to decide for myself how to do things in my daily life (R),” and “In my daily life, I frequently have to do what I am told (R).” The competence items included statements such as “People I know tell me I am good at what I do,” and “Most days I feel a sense of accomplishment from what I do.” The relatedness items included statements such as “I get along with people I come into contact with” and “I really like the people I interact with.” Responses ranged from 1 (not at all true) to 5 (very true). The questions were also provided to the participants in the translated form suggested by Korean research that developed and validated the Korean version of the BPNS scale (Lee and Kim, 2008). Subsequently, confirmatory factor analysis (CFA) was conducted to examine the factor structure of the basic psychological needs. Based on the analysis, the factor loading of each item appeared to be above 0.6, which is deemed acceptable. According to previous research, standardized factor loadings should surpass a minimum threshold of 0.5, with values above 0.6 considered indicative of a good level (Hair et al., 2006; Shek and Yu, 2014). The model fit indices also fell within the permissible range (CMIN/df = 1.776, CFI = 0.986, RMSEA = 0.049, TLI = 0.979).

**Table 1:** Results of confirmatory factor analysis.

Constructs	Indicator	Standard Loading	CMIN/df	CFI	RMSEA
	Autonomy 1	0.75			
	Autonomy 2	0.89			
	Autonomy 3	0.80			
	Competence 1	0.82			
BPNS	Competence 1	0.75	1.77	0.98	0.04
	Competence 3	0.77			
	Relatedness 1	0.75			
	Relatedness 2	0.73			
	Relatedness 3	0.80			
					
	Intrinsic 1	0.56			
	Intrinsic 2	0.75			
Motivation	Intrinsic 3	0.62	1,34	0.99	,03
	Extrinsic1	0.55			
	Extrinsic2	0.76			
	Extrinsic3	,81			

All standard loadings were significant at *p* < 0.001.

Intrinsic motivation refers to engaging in an activity for the inherent enjoyment and satisfaction derived from the activity itself rather than external factors (Deci, 1975). Intrinsically motivated individuals are known to place more emphasis on factors such as personal enjoyment and fun rather than external factors like rewards or recognition (Pelletier et al., 1995). However, most existing measures of intrinsic and extrinsic motivation have been developed primarily within the context of sports or work environments (Pelletier et al., 1995; Tremblay et al., 2009), with limited scales designed for digital gaming contexts. Therefore, in this study, three items from a sports video game motivation scale were adapted and modified based on theoretical exploration. The items were designed for participants to respond on a scale of 1 (strongly disagree) to 5 (strongly agree). For example, items included “I play the game because it is interesting to me,” “The game is the most enjoyable leisure activity for me,” and “The game is one of the ways I have fun with my time.”

Extrinsic motivation, on the other hand, is associated with engaging in an activity for external factors such as rewards obtained from winning or other external incentives. In sports or games, extrinsic motivation may manifest in a form that emphasizes winning (prestige and rewards) over the inherent enjoyment or interest in the activity itself (Pelletier et al., 1995). Considering this, the present study utilized a modified version of a sports video game motivation scale based on previous research on extrinsic motivation (Pelletier et al., 1995; Cianfrone et al., 2011). The items were adapted and modified for measurement, including statements such as “I want to show others the high rank and rewards I have achieved,” “I play the game solely to win,” and “Winning is the most valuable aspect in the game.” These items were also rated on a scale of 1 (strongly disagree) to 5 (strongly agree). The items of the intrinsic motivation and extrinsic motivation scales were translated into Korean by the researchers to fit the context of the game. Subsequently, these questions were reviewed and revised by two peer researchers before being finalized. The finalized translated version was then provided to the participants. Following data collection, a CFA was performed for both intrinsic and extrinsic motivation (see Table 1). The results indicated that for each construct, one item had a standardized factor loading above 0.5, while the remaining items exhibited loadings greater than 0.6, thereby confirming that the levels were within an acceptable range. Additionally, the model fit was confirmed to be within an acceptable range (CMIN/df = 1.314, CFI = 0.994, RMSEA = 0.031, TLI = 0.988).

## 4 Results

We conducted reliability and validity tests on the measured items. Firstly, we examined the average variance extracted (AVE), composite reliability (CR), and Cronbach’s alpha values (See Table 2). The analysis results indicated that all items had CR values of 0.7 or higher and AVE values of 0.5 or higher, which demonstrated the suitability of the analysis (Fornell and Larcker, 1981; Bagozzi and Yi, 1988). Additionally, all items showed Cronbach’s alpha values of 0.6 or higher. While a reliability of 0.7 or higher is generally considered desirable (Nunnally, 1994), a reliability of 0.6 or higher is also acceptable in research (Churchill, 1979). Following that, we performed correlation and discriminant validity tests (See Table 3), and the squared correlations between each pair of variables were smaller than the AVE values, indicating acceptable discriminant validity (Fornell and Larcker, 1981).

**Table 2:** Reliability of constructs.

	# of items	Mean	SD	*α*	AVE	CR
Game cheating	4	2.16	1.06	0.91	0.66	0.88
Autonomy	3	3.50	1.36	0.85	0.52	0.76
Competence	3	4.58	1.05	0.80	0.52	0.76
Relatedness	3	4.79	1.16	0.80	0.53	0.77
Intrinsic motivation	3	3.21	0.86	0.73	0.60	0.82
Extrinsic motivation	3	3.71	0.67	0.68	0.51	0.76

**Table 3:** Discriminant validity analysis.

	1	2	3	4	5	6
Cheating	**0.66**					
Autonomy	0.38	**0.52**				
Competence	0.02	0.04	**0.52**			
Relatedness	0.08	0.05	0.47	**0.53**		
Intrinsic motivation	0.08	0.05	0.00	0.00	**0.51**	
Extrinsic motivation	0.09	0.04	0.18	0.29	0.02	**0.51**

Correlations are below the diagonal and AVE is presented on the diagonal in bold.

Subsequently, we conducted structural equation modeling using AMOS 22.0, and the model fit indices demonstrated good fit: incremental fit index (IFI) = 0.948, Tucker-Lewis’s index (TLI) = 0.933, comparative fit index (CFI) = 0.947, and root mean square error of approximation (RMSEA) = 0.051.

**Figure 1:**
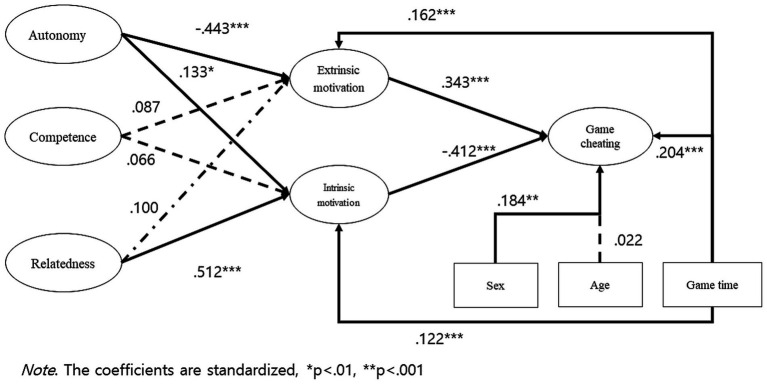
Structural equation model.

The findings of the analysis are presented in Figure 1 and Table 4. The analysis results showed that autonomy was negatively related to extrinsic motivation (*β* = −0.443, *p* < 0.001) and positively related to intrinsic motivation (*β* = 0.133, *p* < 0.05). Relatedness showed no significant relationship with extrinsic motivation (*β* = 0.100), but it had a positive relationship with intrinsic motivation (*β* = 0.512, p < 0.001). On the other hand, competence showed no significant relationship with intrinsic motivation and extrinsic motivation (*β* = 0.066 and − 0.87, respectively). Thus, H2a and H2b were rejected.

**Table 4:** Hypothesis test results.

	*B*	*β*	C.R.	Results
(H1a) Autonomy → Intrinsic motivation	0.052*	0.133	1.969	Accepted
(H1b) Autonomy → Extrinsic motivation	−0.261***	−0.443	−5.364	Accepted
(H2a) Competence → Intrinsic motivation	0.037	0.066	0.603	Rejected
(H2b) Competence → Extrinsic motivation	0.073	0.087	0.837	Rejected
(H3a) Relatedness → Intrinsic motivation	0.257***	0.512	4.131	Accepted
(H3b) Relatedness → Extrinsic motivation	0.075	0.1	0.932	Rejected

*p < 0.05; **p < 0.01; and ***p < 0.001.

Furthermore, extrinsic motivation was positively related to game cheating (*β* = 0.343, *p* < 0.001), while intrinsic motivation was negatively related to game cheating (*β* = −0.412, *p* < 0.001). Gender was found to be positively related to game cheating, indicating that females were more likely to engage in game cheating (*β* = 0.184, *p* < 0.005). Game time was positively related to extrinsic motivation, intrinsic motivation, and game cheating (*β* = 0.162, 0.122, and 0.204, respectively).

## 5 Discussion

### 5.1 Findings of the study

The first finding of this study confirmed the influence of basic psychological needs on intrinsic and extrinsic motivation. Specifically, this study is significant as it empirically confirmed the relationship between basic psychological needs and game usage motivation, which had been relatively under-researched compared to other fields, such as sports. Particularly, this study, unlike some research that employed SDT and focused on basic psychological needs within an in-game context (Ryan et al., 2006), explored the impact of basic psychological needs on game usage motivation. While definitive conclusions are challenging due to the need for further research, the findings suggest that a deficiency in real-life needs can distinctively influence the formation of motivation toward game content through various processes, including compensatory mechanisms (Fernandez de Henestrosa et al., 2023). Moreover, the results of this study, which centered on players of team-based online competitive games, only partially aligned with the relationship between basic psychological needs and intrinsic/extrinsic motivation as delineated in SDT. Firstly, autonomy, one of the basic psychological needs, showed a positive relationship with intrinsic motivation, while it exhibited a negative relationship with extrinsic motivation. This aligns with prior research suggesting that autonomy is one of the crucial psychological needs in shaping motivation (Deci and Ryan, 2000). According to previous studies, individuals who perceive the ability to choose a specific behavior on their own tend to prioritize their interest or enjoyment when participating in a specific activity (Deci and Ryan, 2000; Ryan and Deci, 2006; Dysvik et al., 2013). Conversely, in situations where autonomy is lacking or infringed upon (e.g., external rewards, negative feedback), intrinsic motivation tends to be relatively suppressed (Guay et al., 2001). In this context, the results of this study support the fact that individuals with high autonomy are more likely to choose games for their interest or enjoyment, while those with lower autonomy are more likely to participate in games to achieve victory and the corresponding rewards, rather than prioritizing personal interest or enjoyment.

On the other hand, relatedness significantly influenced intrinsic motivation but did not affect extrinsic motivation. This aligns with prior research suggesting that the stronger and more stable the bond with others, the greater the possibility of promoting intrinsic motivation (Deci and Ryan, 2000). Meanwhile, relatedness appeared to be unrelated to extrinsic motivation. This is consistent with the research findings that the fulfillment of relatedness does not significantly predict identified regulation motivation, a type of extrinsic motivation in sports (Matsumoto and Takenaka, 2022). The study showed that while relatedness significantly affects autonomous and controlled motivations for women, for men, the fulfillment of relatedness only influenced intrinsic motivation. In a similar context, another study focused on physical activities in children and adolescents reported that the influence of relatedness satisfaction on intrinsic and extrinsic motivation was only evident in grade 5 girls (Huhtiniemi et al., 2019). The same study also discovered that when conducted with grade 8 students, the influence of relatedness on motivation formation did not appear in both genders. It was suggested that this might be because having an interest in physical activities is more related to having a sense of agency or an opportunity to prove one’s competence than being part of a safe group. Given these facts, the study’s results can be interpreted to suggest that while the bond with others has a somewhat positive effect on enjoying the game and having fun and interest in the activity, it does not suppress or stimulate extrinsic motivation.

Conversely, competence did not influence any form of motivation, a result that contrasts with the general research findings that competence is a key factor in forming motivation. However, some studies have reported that competence might not affect intrinsic motivation on its own. For instance, a study conducted on 1,254 service organization employees in Norway reported that while competence did not influence motivation formation, in situations of high autonomy, competence showed a significant correlation with intrinsic motivation (Dysvik et al., 2013). This suggests that the influence of competence on motivation can appear through interactions with other needs or psychological factors. Additionally, the absence of the influence of competence could potentially be related to trait or psychological factors that are deeply linked to competence. For example, self-esteem, a psychological factor associated with competence, may induce individuals to behave more defensively to maintain a competent self-image in certain contexts or to selectively participate in specific activities (Ryan and Deci, 2000c). While it is difficult to make definitive claims based on the current study’s findings, these facts suggest that the effect of competence could be moderated by individual differences in psychological factors.

The second finding is the empirical verification of the impact of intrinsic and extrinsic motivations on attitude about game cheating. Considering the fact that attitudes justifying dishonesty positively can promote negative behaviors (Jordan, 2001), this is an important finding that, within the context of digital games, inhibitions regarding potentially harmful actions can occur depending on the type of motivation. The results indicated that people motivated by interest or enjoyment of the game tend to view game cheating negatively. Moreover, motivation from game victory and rewards from victory is positively associated with game cheating. This is consistent with previous research suggesting that while intrinsic motivation suppresses dishonest behavior or cheating, extrinsic motivation can promote it (Kanat-Maymon et al., 2015; Pulfrey et al., 2019; Park, 2020).

The influence of extrinsic motivation on game cheating is particularly noteworthy. Many online competitive games employ strategies to encourage tournament participation by offering rare rewards to players who win more within a limited time frame. However, according to the cognitive evaluation theory, a sub-theory of self-determination theory, external factors such as evaluation, time limits, and rewards can reduce autonomy, alter the perceived locus of causality, and weaken intrinsic motivation (deCharms, 1968; Gagné and Deci, 2005). This suggests that game reward systems or marketing strategies emphasizing rewards or honors associated with winning may suppress an individual’s intrinsic motivation toward a game (Song et al., 2013; Schöber and Stadtmann, 2022).

The influence of extrinsic motivation on game cheating may also be related to the medium-specific characteristics of digital games. For instance, competitive environments like sports have communities and institutions that require compliance with fair play norms, thus potentially easing the value-based conflict between ‘win at all costs’ and ‘sportsmanship’ over time (Bardi and Schwartz, 2013). In contrast, in the anonymous environment of digital games, players can easily overlook or forget moral norms and punitive bodies that could restrain them when faced with value-based conflict, thus leaving fewer options to caution against and control ‘certain but immoral methods of winning.’ For example, the influence of gaming communities in suppressing game cheating can vary depending on the social ties to gaming communities and evolving gaming norms (Chen and Ong, 2018) and could even be further promoted by community norms encouraging game cheating (Wu and Chen, 2018). In such anonymous environments where moral feedback is difficult, players may more readily resort to game cheating if they judge that the reward for winning is greater than the potential loss from punishment (Chen and Wu, 2015; Schöber and Stadtmann, 2022). Therefore, the extrinsic motivation for gaming activities is closely linked to game design and competitive structures, which highlights the sensitivity and complexity of addressing the problem of game cheating in competitive games. Measures to alleviate the pressure inducing extrinsic motivation could inadvertently lead to decreased competition participation.

On the other hand, this study found a positive correlation between females and game cheating, which contrasts with previous studies that suggest men are more likely to engage in behaviors such as academic cheating than women (Calabrese and Cochran, 1990). However, some studies that focused on game cheating reported that the influence of group identification could make women more likely to cheat than men (Chen and Wu, 2015). According to previous research, men are more likely to resort to cheating for individual glorification, such as rewards or displaying honor (Evans et al., 1993). But Chen and Wu’s study suggests that women, who react more sensitively to relationships and social norms than men, can engage in dishonest acts for group norms or social values. While this study’s model does not include factors related to the gaming community and social identity, it cannot be definitively said that the correlation between women and game cheating could be due to communal characteristics. Another possibility is that the negative gaming culture and biases among male gamers may have influenced the cheating behavior of female gamers. According to prior research, there exists a gender stereotype in competitive online gaming culture that women are less “hard-core” gamers and have inferior gaming skills (Lopez-Fernandez et al., 2019). These stereotypes may inhibit or discourage female gamers from mastering or engaging in the game, and players who are heavily exposed to these biases might consider cheating as one of the means to escape gender-based criticism. Lastly, the current findings should be interpreted cautiously as they may be due to the unbalanced recruitment of male and female participants. Further follow-up research is needed.

### 5.2 Theoretical and practical contributions

This study has theoretical significance since it examines the causes of game cheating in the context of self-determination theory. While self-determination theory has been used to understand dishonest behaviors and cheating in academics and sports, its application to game cheating has been rare, and studies on game cheating have mainly been limited to the development of detection technology and other technical solutions. However, this study empirically measured how the basic psychological needs (autonomy, competence, relatedness) proposed by self-determination theory and types of motivation (intrinsic, extrinsic) relate to game cheating. The analysis found that attitudes toward game cheating can differ based on the type of motivation for the game. Particularly in this process, autonomy was confirmed as an important variable in motivation formation. Moreover, contrary to general research results, it was found that the effect of competence on game cheating is limited. This means that analyzing the player’s psychological factors must accompany understanding the relationship between need fulfillment and motivation formation more deeply. These facts suggest the need to construct a model that integrates variables derived from self-determination theory and other psychological variables reported related to cheating in future game cheating studies.

Moreover, the study’s results can help establish practical alternatives for inhibiting game cheating. Firstly, this study shows that enhancing a player’s autonomy can potentially promote intrinsic motivation while reducing extrinsic motivation. Considering the results of previous studies suggesting that autonomy activates intrinsic motivation (Gagné and Deci, 2005), from the game design’s perspective, ensuring various choices for players can assist in activating intrinsic motivation and inhibiting cheating. For instance, game companies need to focus on implementing mechanisms for efficient victories and various choices and mechanisms that consider players’ interests and individualities to enhance their autonomy. Also, in the long run, building campaigns and additional systems to make players choose and follow fair play norms by themselves, rather than being passively moved by warnings or punishments by the system, would be useful.

In addition, the positive correlation between game cheating and extrinsic motivation provides relevant implications for game design and reward system design. According to the results of this study, excessive motivation for victory and the rewards that come with victory can lead to game cheating. This suggests that game design suppressing excessive extrinsic motivation could help inhibit cheating. For instance, game companies could adjust the level of rewards for winners or set up buffer systems to alleviate the mental burden and loss from defeat. Of course, in games with competitive structures, determining winners and losers is an inevitable result, and it might be strictly impossible to exclude marketing strategies that use rewards and honor to encourage competitive participation in commercial games. Nonetheless, there is a need to improve irrational reward systems, where winners monopolize rare rewards, to a realistic level. Along with this, as currently introduced in League of Legends, implementing systems that provide separate rewards to users who demonstrate fair play, or enhancing the value of fair play scores, could be alternatives. Moreover, because game cheating often occurs from the perception that the benefits of dishonest behavior exceed the loss (Schöber and Stadtmann, 2022), even if the motivation for rewards is formed, strengthening the level of punishment to prevent it from leading to immoral behavior, and notifying players of this, could also be helpful.

Lastly, the influence of psychological needs and intrinsic motivation observed in this study provides useful guidelines in educational and policy aspects. For instance, eSports player training institutions could add programs to check basic psychological needs, in addition to education about game ethics, to prevent and inhibit players’ immoral game behaviors. Also, in-game education for the public, such as by game literacy organizations, could guide and avoid the damage that a ‘win at all cost mentality and excessive obsession with external rewards’ could bring and set up programs to enhance the fun and interest inherent in-game activities. Considering also the recent trend of a significantly expanding generation of game users, refined development of educational methods is needed to teach game ethics, the attitudes and moral qualities to have while playing competitive games, and above all, the joy of playing the game itself.

### 5.3 Limitations

Despite the discoveries about game cheating in this study, it has some limitations. The first limitation is that it did not comprehensively deal with various types of motivation. Furthermore, we did not examine the mediating effect between basic psychological needs, motivation, and game cheating. This implies that our study employed the Self-Determination Theory (SDT) in a limited manner to investigate attitudes toward game cheating. For instance, extrinsic motivation ranges from external regulation, where the level of autonomy is lowest, to integrated regulation, which has a relatively high level of autonomy, and there also exists amotivation apart from intrinsic motivation (Gagné and Deci, 2005; Ryan and Deci, 2017). However, this study had the limitation of using only two types—intrinsic motivation and extrinsic motivation—due to the lack of motivation scales suitable for the digital game environment and the absence of preceding studies. Therefore, future research needs to comprehensively explore the relationship between gaming misconduct, various types of motivation, and basic psychological needs.

The next limitation is that the research did not broadly cover environmental, moral, and psychological factors that could influence game cheating outside of self-determination theory. For instance, organizational norms and culture, awareness of moral rules, and perceived justice regarding cheating punishment rules could significantly influence attitudes toward cheating (Lemons and Seaton, 2011; Oberman et al., 2021). Particularly, as verified in the post-hoc study, elucidating the relationships that multiple variables associated with cheating have with basic psychological needs and types of motivation is essential in advancing more in-depth research. Therefore, further studies must set up and analyze an integrated model for game cheating, supplementing the self-determination theory model.

Furthermore, this study involved translating some scales by the researcher and having them reviewed by peer researchers, yet it did not undergo a separate academic procedure to minimize errors in the translation process. Moving forward, there will be a need to conduct research using scales that are reliable, considering cultural or linguistic contexts through a rigorous process.

Digital game cheating is a crucial problem threatening the spread of game culture and the growth of the eSports industry. However, as proven in sports and other fields, solving dishonesty and cheating problems is deeply connected with ethical issues and psychosocial factors; hence, a solely technical response has its limits. Therefore, in the future, there need to be more attempts to clarify the influencing factors leading players to cheat through self-determination theory and various psychosocial theories and concepts and seek comprehensive response measures based on these.

## Data availability statement

The raw data supporting the conclusions of this article will be made available by the authors, without undue reservation.

## Ethics statement

The studies involving humans were approved by the Konkuk University Institutional Review Board. The studies were conducted in accordance with the local legislation and institutional requirements. The participants provided their written informed consent to participate in this study.

## Author contributions

SL: Conceptualization, Methodology, Writing – original draft. EL: Methodology, Supervision, Writing – review & editing, Writing – original draft. DK: Formal analysis, Methodology, Writing – review & editing. JK: Methodology, Writing – review & editing.
